# Occurrence and human exposure assessment of PFAS in river and groundwater around a closed fluorochemical plant in China

**DOI:** 10.1038/s41598-025-01128-6

**Published:** 2025-05-09

**Authors:** Chunyan Xu, Yijing Yang, Haibo Ling, Chuan Yi, Xiangpu Zhang, Ruowen Zhang

**Affiliations:** 1 Hubei Academy of Environmental Sciences, Wuhan, Hubei People’s Republic of China; 2 Hubei key Laboratory of Pollution Damage Assessment and Environmental Health Risk Prevention and Control, Wuhan, Hubei People’s Republic of China

**Keywords:** PFASs, Closed fluorochemical facility, Surface water, Groundwater, Human exposure, Environmental sciences, Environmental impact

## Abstract

Studies have demonstrated that point source emissions constitute the main direct source of PFASs in water. However, if production/usage and emission from a specific point are stopped, does the point source still present a threat to surrounding waters? In this study, the occurrence and potential human exposure to 17 PFASs in the surrounding ambient river and ground/drinking water within a 13 km around the facility were assessed. Of the 17 PFASs analyzed, 11 were frequently detected in river and groundwater samples, with perfluorobutane sulfonate (PFBS) (36.8−11462.9 ng/L), perfluorobutyric acid (PFBA) (below the detection limit (BDL)−4789.8 ng/L) and perfluorohexane sulfonate (PFHxS) (3.3−3549.0 ng/L) exhibiting the highest concentrations. Prevalence of short-chain PFASs was observed in both river and groundwater. The spatial distribution pattern showed that locations near the facility exhibited higher PFASs concentrations. The seasonal distribution pattern indicated that the PFASs concentration in river water during the wet season was higher than that during the dry season. However, the seasonal distribution in groundwater was unexpectedly the opposite to that in river water. Nevertheless, the major health risk of PFASs is primarily attributed to the presence of perfluorooctane sulfonate (PFOS) and perfluorooctanoic acid (PFOA) with maximum hazard quotients of 6.9 × 10^2^ and 1.0 × 10^3^ and 2.4 and 3.6 for adults and toddlers, respectively. Thus, the potential threat of the closed fluorochemical manufacturing plant to the surrounding waters cannot be ignored.

**Supplementary Information:**

The online version contains supplementary material available at 10.1038/s41598-025-01128-6.

## Introduction

 Per- and polyfluoroalkyl substances (PFASs) are a large group of chemicals which consist of a fully (per) or partly (poly) fluorinated carbon chain connected to different functional groups^1^. Based on the length of the fluorinated carbon chain, short and long chain PFASs can be distinguished. The wide use of PFASs in various industries and consumer products has resulted in their pervasive presence in the environment. PFASs have been identified in biotic and abiotic matrices, including soil^2^, water^3^, air^4^, and wildlife^5^, and even in human biosamples, such as urine^6^, serum^6^ and breast milk^7^. The growing concern regarding the bioaccumulation and potential toxicity of PFASs to humans has promoted the worldwide restriction/elimination of the production and usage of PFASs. For example, perfluorooctane sulfonic acid (PFOS), perfluorooctanoic acid (PFOA) and perfluoro-hexane sulfonic acid (PFHxS) were all included in the Stockholm Convention in 2009, 2019 and 2022, respectively^8–10^.

China began to produce PFASs around the year 2000, but before 2003, the quantity of PFASs produced in China was relatively small (annual production < 50 tons)^11^. The phasing out of PFOS in Japan, the U.S., and Western European countries^12^ has promoted the development of PFOS production in developing countries, including China. By 2012, the annual production volume of perfluorooctane sulfonyl fluoride (POSF) in China reached approximately 170 tons. In 2015, To promote PFOS elimination, the Chinese government and environmental protection department have adopted a series of measures, including the implementation of the China PFOS Priority Industry Reduction and Elimination Reserve Project, in which all PFOS uses not listed as exempted or acceptable are banned. Afterward, the production of PFOS in China sharply declined. With the assistance of the World Bank and the Chinese government, certain manufacturers have started to shift their production towards short-chain substitutes, such as perfluorobutane sulfonyl fluoride (PBSF).

It is generally assumed that point source, particularly the production and usage of fluorochemicals, as well as the manufacturing of products containing PFASs, due to the significant emissions of PFAS waste^13^. For example, studies conducted at fluorochemical production facilities in France^14^ and China^11^, as well as firefighter training areas^15^, have demonstrated that PFASs can be released into nearby water. However, if production and emission have ceased at a given site, could the facility still be a source of PFASs and pose a potential risk to the environment? No pertinent findings have been reported yet. Considering the persistence of PFASs in the environment, the risk of closed fluorochemical facilities to ambient water cannot be ignored.

In this study, we concentrated on a fluorochemical production facility that was previously the world’s largest POSF production facility but has been closed for two years. Since PFASs are not included in China’s pollution discharge limits or environmental monitoring standards, the pollution status in the surrounding area remains unknown. Here, the presence of 17 PFASs in the ambient water of the facility was characterized over the course of one year. The monitored matrices encompassed river water and groundwater, which serves as untreated alternative drinking water for the majority of rural inhabitants in the area. The primary aim of this research was to investigate the PFASs pollution status of ambient water near the fluorochemical production plant as supplemental data to evaluate the impact of closed manufacturing facilities on the surrounding water. Additionally, the study assessed the exposure patterns by considering the drinking water sources utilized by the local population and evaluated the levels of human exposure resulting from the consumption of drinking water.

## Materials and methods

### Chemicals and standards

A commercially available stock solution containing a mixture of 17 native PFASs and mass-labeled internal standards was procured from TMstandard (China). The concentration of the stock solution was 2 µg/mL in methanol. The target PFAS were listed in Table 1. Other reagents included methanol and acetonitrile for HPLC analysis, ≥ 99.9% (Fisher, USA), ammonium hydroxide (25%, China), acetic acid and ammonium acetate (99.7%, China). Solid phase extraction cartridges were purchased from Waters (6 mL, 150 mg, Oasis® WAX). And Milli-Q water (MQ) (Millipore, Billerica, Germany) was used throughout the study.

### Sample site and sampling

The fluorochemical manufacturing facility is situated in Hubei province, central China, which started production in 2004 and have shut down in 2021. During the period from 2004 to 2014, the facility primarily product POSF, perfluorooctanesulfonic acid potassium salt and their derivatives with an annual production of 30 tons through electrochemical fluorination (ECF). In 2017, in response to China’s PFOS elimination regulations, the production shifted to the short-chain substitute such as PBSF, PHxSF and their derivatives, with a scale of 400–500 tons per year. In the year 2021, the facility ceased all production activities.

**Fig. 1:**
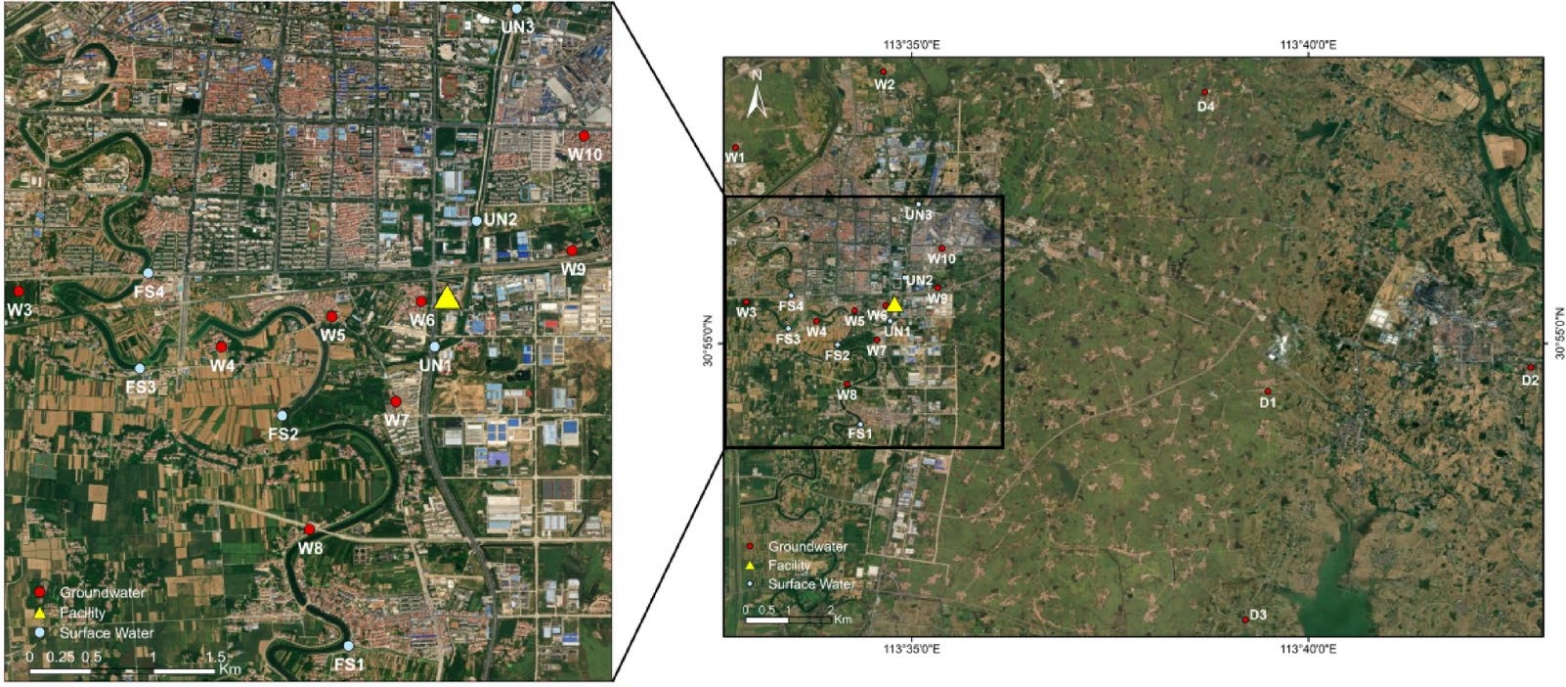
Sampling map for the river and ground water in this study. (The yellow triangle represents the closed fluorochemical production plant). The maps were generated based on an online map using ArcGIS 10.8 (Environmental Systems Research Institute, USA. https://www.esri.com/) and by annotating the sampling locations.

Most sampling sites are located close proximity to the plant. River water and domestic groundwater sampling was mainly carried out within a distance of 5 km from the plant. Sampling sites for public-supply wells (also belong to groundwater) are relatively far away, with a distance of 7–13 km from the factory area. The sampling campaign was conducted at a total of 21 different locations: 4 along the Fushui river tributary (located around the plant, the main tributary of Fushui river, which is the largest surface water of the city and the main water source of agriculture and domestic); 3 along the unnamed river (located adjacent to the plant, the main receiving water body of the plant’s discharge effluent), 10 were domestic well (from the household around the plant) and 4 were public-supply wells (each wells serves a village with a population of 300–500). Figure 1 shows the detailed sampling locations, specific information of each sampling sites can be found in Table S1. Water samples were collected in April 2022 (wet season) and January 2023 (dry season), respectively. One time grab water samples were collected from each location and preserved in 1 L new wide-mouth polypropylene (PP) bottles at a temperature of 4 °C. To prevent contamination, PP bottles were precleaned with ultrapure water, methanol and rinsed with water sample 2–3 times in the field before sampling. Sample duplicates and field blank were collected along with the samples. The samples were subsequently analyzed within a week of collection.

The water sample extraction method was adapted from previous studies^16^. Briefly, 500 mL water samples were filtered with a 0.7 μm GF/F membrane (CNW, Germany). The WAX cartridges were preconditioned with 6 mL of a 2% (v/v) NH_4_OH solution in methanol followed by 6 mL methanol and finally 6 mL ultrapure water. Water samples were then spiked with 2 ng of an internal standard (including ^13^C-PFOA and ^13^C-PFOS, with CAS number 960315-48-4 and 960315-53-1, respectively) and loaded onto the cartridge at a rate of 3 mL/min-5 mL/min. After all the samples had passed through the cartridges, the cartridges were cleaned with 8 mL of an ammonium acetate buffer (25 mM). The cartridges were dried under vacuum for 10 min and washed with 8 mL methanol. The target fraction was eluted with 6 mL 2% (v/v) NH_4_OH in methanol and collected in 15 mL PP tubes. The eluent was evaporated to near dryness with a nitrogen stream and then reconstituted with 1 mL pure methanol. Finally, the eluent was filtered into 1.5 mL amber vial through nylon filter.

### Instrumental analysis

PFASs were analyzed with an Agilent 1290 LC (Agilent Technologies, Germany) coupled to an AB 3500 Triple Quadrupole MS (AB SCIEX, Singapore) in negative ionization mode. 10 µL extract was injected into a ChromCore C18 column (Amerigo Scientific, 2.1 × 150 mm, 3 μm) at a flow rate of 0.3 mL/min and the column temperature was constant at 40℃. The mobile phase A was 2 mM ammonium acetate aqueous solution, and the mobile phase B was methanol/acetonitrile (volume ratio: 1:1). A solvent gradient was programmed as follows: 5–50% B at 0–1.5 min, increasing to 50–95% B at 1.5–3 min, 95% B held until 7 min, and decreasing back to 5% B by 8 min. The corresponding instrument parameters of the target PFASs are shown in the Table S2.

### Quality assurance and quality control (QA/QC)

In this study, all experimental containers were made of polypropylene. Rigorous quality control measures were implemented throughout the various stages of sample collection, transportation, pretreatment, and quantitation analysis. Except for the water samples, the sampling process also included one field blank and one transportation blank (ultrapure water). In the pretreatment process, procedure blanks, laboratory parallel and matrix spiked sample were set for each batch of samples (every 10 samples). During instrumental analysis, one solvent blank (methanol) was also introduced to every 10 samples. No target compounds were detected in any of the blanks. To assess the linearity, seven-point calibration curves were prepared with native concentrations ranging from 0.1 to 50 µg/L, and the correlation coefficients (R) of the calibration curves for all target PFAS were above 0.99 (Table 1). For all compounds, concentrations of the target analytes in all samples were corrected for the surrogate standard recoveries. The reporting limits (RLs) were defined as half of the lowest point of the calibration curve and the reporting limits were 0.1 ng/L in filtered water for all target analytes. The recoveries of matrix spiking ranged from 71 to 117%, and the relative standard deviations (RSDs) for repeatability were all below 30%, indicating good precision ^13^C_4_-PFOA and ^13^C_4_-PFOS recoveries ranged from 51.9 to 90.3% and 53.4 to 96.4% with average values of 65.5% and 72.7%, respectively.

**Table 1:** Abbreviation, calibration curve, correlation coefficients, RLs and recovery of 17 target PFASs.

Compound	Molecular formula	calibration curve	Correlation coefficient	RLs (ng/L)	Matrix spiked recovery (%)
Perfluorobutanoic acid (PFBA)	CF_3_(CF_2_)_2_COOH	*y* = 1.4762*x* + 0.01663	0.99791	0.1	106%
Perfluoropentanoic acid (PFPeA)	CF_3_(CF_2_)_3_COOH	*y* = 1.17839 *x* + 0.00792	0.99760	0.1	101%
Perfluorohexanoic acid (PFHxA)	CF_3_(CF_2_)_4_COOH	*y* = 1.03485*x*−0.00225	0.99844	0.1	87%
Perfluoroheptanoic acid (PFHpA)	CF_3_(CF_2_)_5_COOH	*y* = 1.51518*x* + 0.04649	0.99541	0.1	97%
Perfluorooctanoic acid (PFOA)	CF_3_(CF_2_)_6_COOH	*y* = 2.17349*x* + 0.25486	0.99574	0.1	108%
Perfluorononanoic acid (PFNA)	CF_3_(CF_2_)_7_COOH	*y* = 1.30073x + 0.08165	0.99816	0.1	101%
Perfluorodecanoic acid (PFDA)	CF_3_(CF_2_)_8_COOH	*y* = 2.2392*x* + 0.08062	0.99856	0.1	98%
Perfluoroundecanoic acid (PFUnDA)	CF_3_(CF_2_)_9_COOH	*y* = 2.08876*x* + 0.03041	0.99582	0.1	106%
Perfluorododecanoic acid (PFDoDA)	CF_3_(CF_2_)_10_COOH	*y* = 1.64996*x* + 0.01252	0.99763	0.1	95%
Perfluorotridecanoic acid (PFTrDA)	CF_3_(CF_2_)_11_COOH	*y* = 0.96514*x* + 0.02118	0.99938	0.1	97%
Perfluorotetradecanoic acid (PFTeDA)	CF_3_(CF_2_)_12_COOH	*y* = 1.46271*x* + 0.02459	0.99984	0.1	93%
Perfluorohexadecanoic acid (PFHxDA)	CF_3_(CF_2_)_14_COOH	*y* = 1.10038*x* + 0.00548	0.99886	0.1	107%
Perfluorooctadecanoic acid (PFODA)	CF_3_(CF_2_)_16_COOH	*y* = 0.27209*x* + 0.01107	0.99580	0.1	117%
Perfluorobutane sulfonate (PFBS)	CF_3_(CF_2_)_3_SO_3_H	*y* = 1.31519*x* + 0.09948	0.99847	0.1	71%
Perfluorohexane sulfonate (PFHxS)	CF_3_(CF_2_)_5_SO_3_H	*y* = 1.02190*x* + 0.01167	0.99664	0.1	98%
Perfluorooctane sulfonate (PFOS)	CF_3_(CF_2_)_7_SO_3_H	*y* = 1.32954*x* + 0.14665	0.99730	0.1	97%
Perfluorodecane sulfonate (PFDS)	CF_3_(CF_2_)_9_SO_3_H	*y* = 0.86205*x* + 0.00555	0.99907	0.1	88%

### Human health risk assessment

Given the variability in the concentration of PFASs across various geographical locations, the potential human exposure to these substances was discussed in three distinct scenarios: (a) low exposure, (b) median exposure and (c) high exposure which were calculated with the minimum, median and maximum concentration, respectively, detected of each PFAS congeners from all ground/drinking water location. Three exposure scenarios to PFASs for adults and toddlers aged 2–6 years via contaminated drinking water were calculated using the methodology proposed by Zhang et al.^17^. The potential risk to human health through consumption of drinking water was quantified as hazard quotient (*HQ*_*H*_) and calculated as:

1 $$HQH=\frac{{EDI}}{{TDI}}$$

The estimation of the Estimated Daily Intake (*EDI*) involved the consideration of three factors, including the environmental concentration (*EnC*) of each PFAS congener, the intake frequency *(IF*), and the body weight (*BW*) (Eq. (2)). The *IF* values used in the calculation were obtained from the Exposure Factors Handbook of the Chinese Population, which indicated an *IF* of 1.9 L/day for adults and 0.75 L/day for toddlers. The *BW* values used were 61 kg and 16 kg for adults and children, respectively^18^.

2 $$EDI=\frac{{EnC \times IF}}{{BW}}$$

The Tolerable Daily Intake (*TDI*) was calculated using the reference dose (*RfD*) and uncertainty factors (*UF*) according to Eq. (3). The *RfDs* were determined based on ecotoxicological studies involving mammals exposed to PFASs orally. The extrapolation *UF* values used were 3/10 for subchronic/subacute to chronic exposure (*UF*_*S*_), 3 for the conversion of lowest observed adverse effect level (LOAEL) to no observed adverse effect level (NOAEL) (*UF*_*L*_), 3 for intra-species variability (*UF*_*A*_), and 10 for inter-species variability (*UF*_*H*_)^17^.

3 $$TDI=\frac{{RfD}}{{UFs \times UFL \times UFA \times UFH}}$$

## Results and discussion

### Occurrence and distribution of PFASs in river water

The overall PFASs detection frequency (DF) and concentration in the obtained river water samples are summarized in Table 2. The detailed detection status of each PFASs is listed in Table S3. Of the 17 PFASs analyzed, 11 were detected in two river water samples at the detection limit of 0.1 ng/L, with a DF ranging from 25.0 to 100.0%. In the Fushui River, the total PFASs concentration (∑PFASs) ranged from 203.0 to 23118.0 ng/L, with a mean total concentration of 9059.9 ng/L. In the unnamed river, ∑PFASs ranged from 32.8 to 23255.0 ng/L, with a mean total concentration of 5191.9 ng/L. Literature data concerning the PFASs levels in surface water surrounding closed facilities are scarce relative to facilities still in operation. Dauchy et al.^15^ evaluated the occurrence of more than 50 PFASs in surface water near firefighter training areas in France and found that ∑PFASs ranged from 1.0 µg/L to 29.0 mg/L. Tang et al.^19^ reported that the total concentration of 11 PFASs ranged from 13.2 to 34.6 ng/L in landscape lakes around an electroplating factory in China. Wang et al.^11^ measured 9 PFASs in 5 ponds and 2 rivers near a fluorochemical facility in China and found that the mean PFOS, PFOA and PFHxS concentrations were 14.1, 10.0, and 7.8 ng/L, respectively. Pétré et al.^20^ quantified 29 PFASs in stream water near a manufacturing facility in North Carolina, U.S., and found that the concentration ranged from 426 to 3617 ng/L. The PFASs concentration in the river water samples measured in this study was much higher than that reported in other regions of China and the U.S. but lower than that reported in firefighter training areas in France, which may be related to the number of PFASs congers detected in the two studies.

**Fig. 2:**
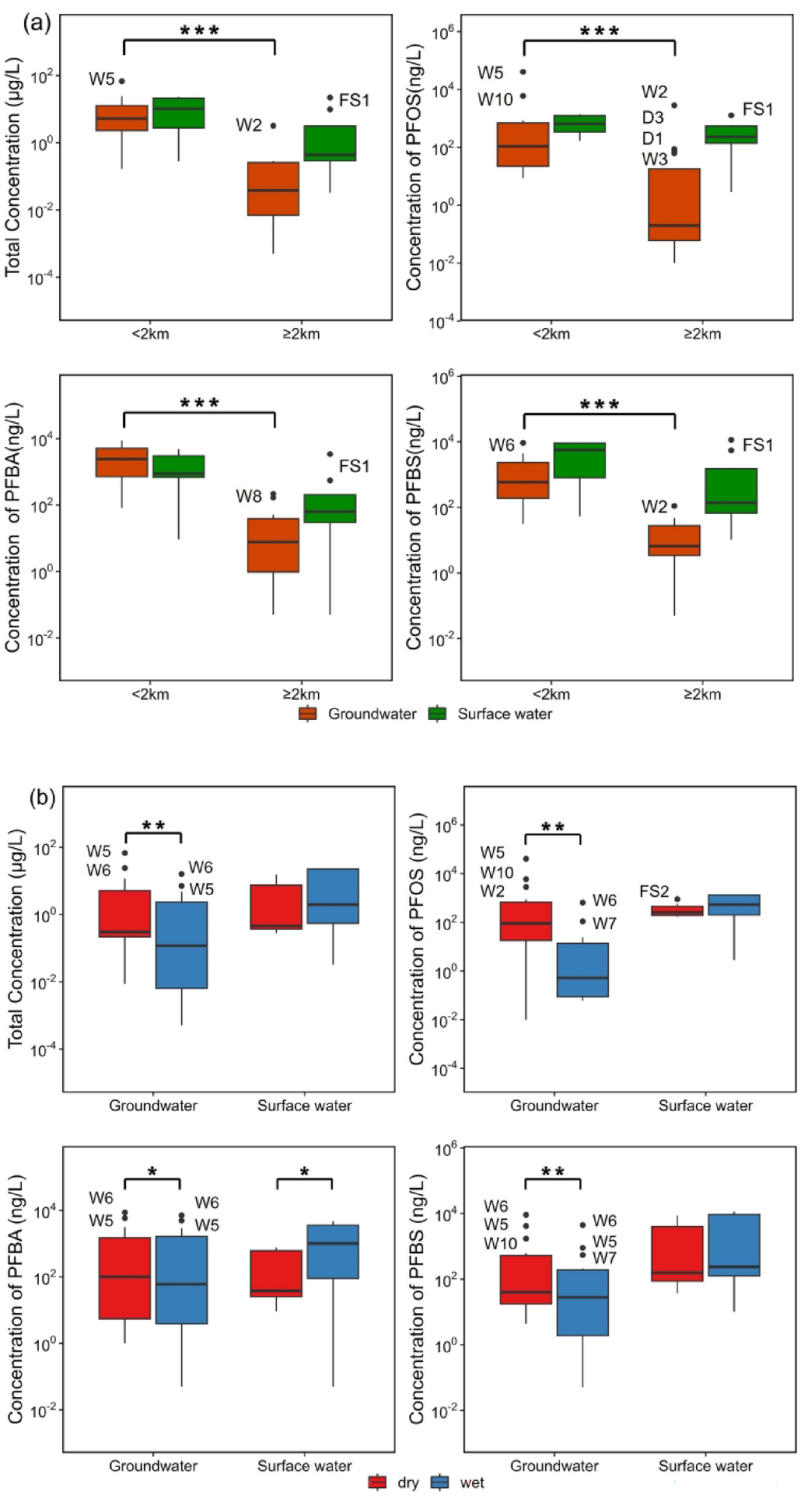
Spatial (**a**) and seasonal (**b**) distribution boxplots of total PFASs, PFOS, PFBA, and PFBS water concentrations. Upper edge of the box, line within the box and lower edge of the box, represents the 75th, 50th, and 25th percentiles. Vertical lines extend from the minimum to the maximum value, while extreme values were labeled with circle. The symbols “*”, “**”, and “***” were used to denote statistical significance at p-values of less than 0.05, 0.01, and 0.001, respectively.

Geographical differences between river water sampling points were evaluated (Fig. 2). Results revealed that samples collected in close proximity to fluorochemical facility (< 2 km) showed comparatively high concentration of ∑PFASs (11528.8 ± 10433.7 ng/L, mean ± SD), PFOS (759.1 ± 540.6 ng/L), PFBA (1827.2 ± 1941.4 ng/L) and PFBS (5092.8 ± 4642.2) than those remote areas (≥ 2 km, ∑PFASs: 4306.6 ± 7973.3 ng/L, PFOS: 382.2 ± 420.8 ng/L, PFBA: 532.0 ± 1186.0 ng/L, PFBS: 2194.9 ± 4198.4 ng/L), but the difference is not statistically significant (*p* > 0.05, Mann-Whitney U test), this could be attributed to the mobility of river. Seasonal trends of PFASs concentrations in river water were also evaluated. The levels of ∑PFASs (4560.4 ± 5995.8 ng/L), PFOS (375.5 ± 271.2 ng/L) and PFBS (2460.5 ± 3469.6 ng/L) obtained during the dry campaigns seemed to offer lower values compared to wet season (∑PFASs: 10243.3 ± 11828.2 ng/L, PFOS: 711.9 ± 625.9 ng/L and PFBS: 4413.3 ± 5390.7 ng/L), but this result lacked statistical significance (*p* > 0.05). While, PFBA levels detected in wet seasons (1877.7 ± 2040.5 ng/L) were statistically higher (*p* < 0.05) than those found in dry season (296.5 ± 344.3 ng/L). Research has demonstrated that the flow level can affect the PFASs concentration in river water^21,22^. In the absence of known nearby PFASs emissions, watersheds with low surface flows generally exhibit relatively high PFASs levels^23^. In contrast, in the presence of a PFASs facility, rainfall may promote contaminant flushing and thus generate higher pollutant concentrations in nearby rivers. In addition, it has been proposed that the timescale of water flushing may be involved in determining the PFASs concentration distribution during different sampling periods^20^. Given the fluorochemical facility ‘s sustained mass production of PBSF from 2017 to 2021, the observed seasonal variation in PFBA concentrations among the 11 detected PFAS analytes could be attributed to three factors: (a) the high concentration of PFBA in ambient environmental compartments (e.g., soil) due to production activity, which combined with the scouring effect of water on ambient pollutants during the wet season, (b) the timescale of water flushing (resulting in higher concentrations in water samples obtained at earlier times), (c) the characteristics of PFBA properties, such as its molecular weight and LogKoc^24^ in comparison to long-chain PFAS (PFOS/PFOA), render it more readily susceptible to leaching from contaminated soil into water bodies.

**Table 2:** Detection frequencies (DF) and range of concentrations (ng/L) of 17 PFASs in river and ground/drinking water.

Analyte	River water				Ground/drinking water			
	The Fushui River (*n* = 8)		The unnamed river (*n* = 6)		The public-supply wells (*n* = 8)		The domestic wells (*n* = 20)	
	DF(%)	Concentration (ng/L)	DF(%)	Concentration (ng/L)	DF(%)	Concentration (ng/L)	DF(%)	Concentration (ng/L)
PFBS	100.0	4439.3 (36.8−11462.9)	100.0	2100.3 (10.3−9624.9)	62.5	8.3 (ND−31.0)	100.0	1128.4 (6.4−9238.5)
PFHxS	100.0	1172.1 (3.3−3349.0)	100.0	713.1 (3.9−3549.0)	50.0	37.9 (ND−160.1)	95.0	494.5 (ND−5999.2)
PFPeA	100.0	131.52 (1.9−372.0)	100.0	75.7 (0.8−338.0)	25.0	0.2 (ND−0.6)	100.0	189.9 (0.1−1259.8)
PFOS	100.0	644.6 (2.8−1392.8)	100.0	409.3 (7.8−1370.8)	50.0	20.8 (ND−85.6)	85.0	2583.7 (ND−40795.8)
PFDS	25.0	1.6 (ND−8.26)	50.0	0.9 (ND−2.81)	0.0	−	10.0	RLs (ND−0.9)
PFBA	100.0	1223.2 (34.1−4789.8)	83.3	905.5 (ND−3726.8)	75.0	1.0 (ND−2.4)	100.0	1922.1 (13.0−8639.5)
PFHxA	100.0	479.7 (7.8−1377.7)	100.0	339.2 (2.9−1462.7)	62.5	1.1 (ND−3.4)	100.0	664.4 (0.1−3351.4)
PFHpA	100.0	285.7 (3.1−743.8)	100.0	180.8 (1.3−836.8)	50.0	0.8 (ND−3.1)	95.0	177.3 (ND−1779.8)
PFOA	100.0	676.7 (8.4−1844.9)	100.0	463.3 (5.2−2333.9)	50.0	3.1 (ND−13.4)	95.0	283.4 (ND−4579.6)
PFNA	100.0	4.4 (0.7−10.6)	100.0	3.2 (0.3−9.1)	50.0	0.1 (ND−0.3)	70.0	2.8 (ND−36.7)
PFDA	25.0	0.71 (ND−2.45)	50.0	0.3 (ND−0.7)	12.5	RLs (ND−0.2)	35.0	3.4 (ND−52.9)
PFUnDA	0.0	−	0.0	−	0.0	−	0.0	−
PFDoDA	0.0	−	0.0	−	0.0	−	0.0	−
PFTrDA	0.0	−	0.0	−	0.0	−	0.0	−
PFTeDA	0.0	−	0.0	−	0.0	−	0.0	−
PFHxDA	0.0	−	0.0	−	0.0	−	0.0	−
PFODA	0.0	−	0.0	−	0.0	−	0.0	−
∑PFASs	−	9059.9 (203.0−23118.0)	−	5191.9 (32.8−23255.0)	−	73.8 (RLs−289.0)	−	7450.4 (20.6−67937.9)

“ND” were defined as concentrations < RLs (0.1 ng/L).

For statistical analysis, all PFASs levels < RLs were counted as half the RLs.

**Fig. 3:**
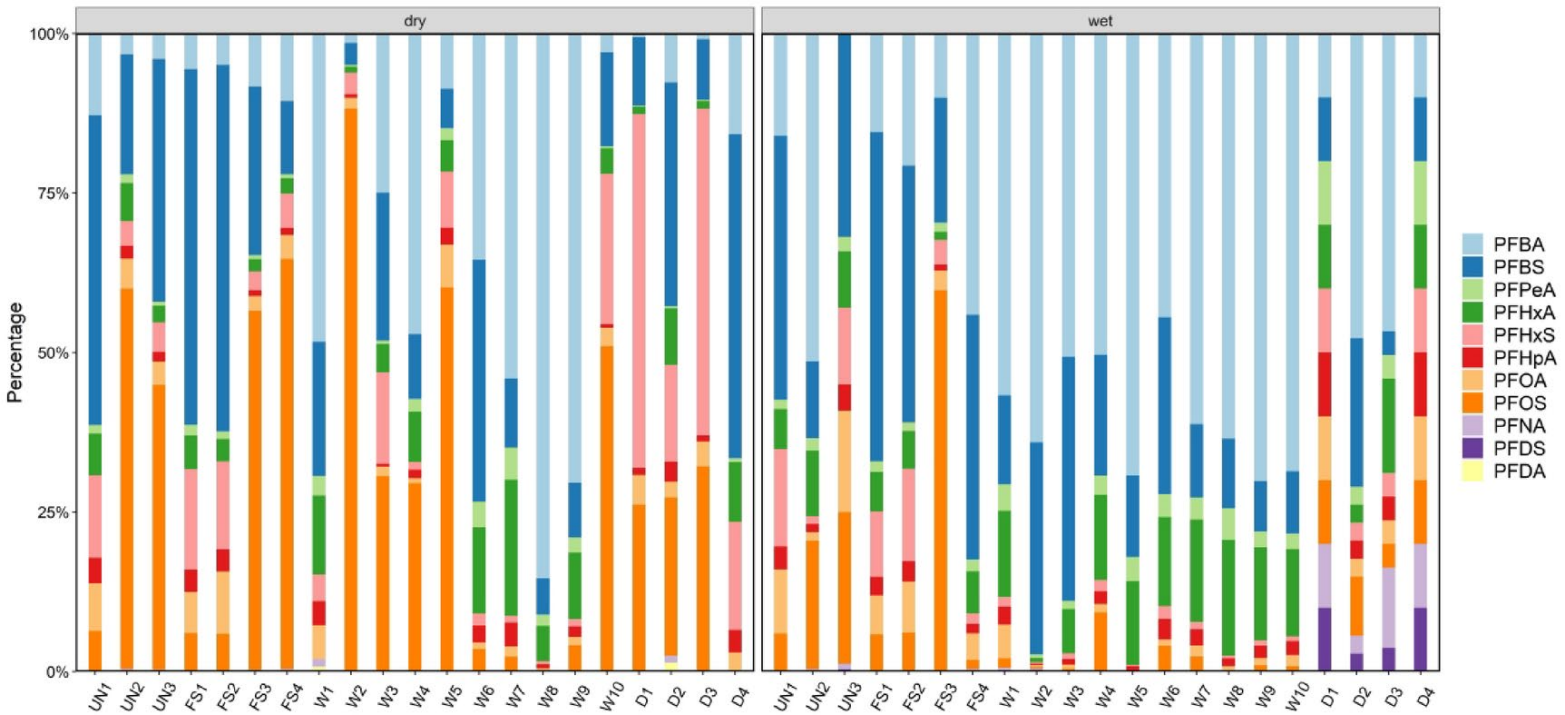
Relative composition of PFASs species in river and ground water of two seasons.

The percentage of each substance at each sample point during the dry and wet seasons is shown in Fig. 3. The dominant PFASs species identified in FS1, FS2, and UN1 during both dry and wet seasons were PFBS (C4), followed by PFHxS (C6) and PFBA (C4), and then the legacy compounds PFOA (C8) and PFOS (C8). These five PFASs species accounted for 49.2%, 13.8%, 12.6%, 8.0% and 6.0% of the total PFASs. However, in FS3, FS4, UN2 and UN3, particularly during the dry season, PFOS emerged as the dominant PFASs species, followed by PFBS and PFBA, and then PFHxS and PFOA. Notably, PFOS accounted for 60.3% of the total PFASs. The sampling sites, FS1, FS2, and UN1, are all located downstream of the river and in close proximity to facility, thus indicating a significant influence of nearby point sources on the concentration of PFASs in the water. Conversely, FS3, FS4, UN2, and UN3 are located upstream of the river, and are relatively less affected by point sources downstream. During periods of low rainfall in the dry season, when the erosive effects of rainwater are diminished, long-chain PFASs with high level of persistence^25,26^ tends to exhibited higher concentration in water. Although the percentage of each PFAS congener varies somewhat in different sampling site and sampling seasons, but in general, the PFAS congener with high concentration in river water, specifically PFBS, PFBA, PFHxS, PFOA and PFOS, were all related to the products that used to be produced by the fluorochemical manufacturing facility. The PFBS, PFOS and PFHxS are the hydrolysis products of PBSF, POSF and PHxSF, respectively, while PFOA may be a by-product of the ECF method of producing PFOS through POSF. The predominant PFASs species detected in this study are consistent with those determined in other studies^11,19^, but the amounts in the two investigated rivers were 29- to 165-fold larger than those previously reported, which could be attributed to the maximum production and emissions of the facility (it was once the largest POSF production facility in the world).

### PFASs in groundwater/drinking water

For many years, groundwater has been an important drinking water source for the residents in most rural areas of the middle reaches of Yangtze River Basin. The sample area, located in this rural context, relies extensively on groundwater, with notable cases where centralized drinking water supplies are sourced from groundwater wells in certain villages. Thus, for those local residents, drinking water remains the predominant exposure pathway to PFASs. The PFASs concentration in groundwater is provided in Table 2. Out of the 17 PFASs, 10 and 11 PFASs were frequently detected in public supply wells and domestic wells, respectively. Six compounds with more than ten carbon atoms (e.g. PFUnDA, PFDoDA, PFTrDA, PFTeDA, PFHxDA, and PFODA) were not detected in any of the groundwater samples, which is consistent with the river water results. Similar to the river water findings, the dominant PFASs species found in groundwater were PFBS, PFBA, PFOS, PFOA and PFHxS. The concentrations of these five compounds in public supply wells and domestic wells ranged from not detected (ND)−31.0 ng/L, ND−2.4 ng/L, ND−85.6 ng/L, ND−13.4 ng/L and ND−160.1 ng/L and from 6.4−9238.5 ng/L, 13.0−8639.5 ng/L, ND−40795.8 ng/L, ND−4579.6 ng/L, and ND−5999.2 ng/L, respectively. Sharma et al.^27^ measured 21 PFASs in groundwater/drinking water in the Ganges River basin in India and found that the predominant compounds were PFHxA, PFHpA, PFPA, and PFOA with concentrations ranging from 0.8−4.9 ng/L, 0.5−3.5 ng/L, less than the method quantification limit (MQL)−2.2 ng/L and < MQL−0.8 ng/L, respectively. McMahon et al.^28^ quantified 24 PFASs in groundwater used as a source of drinking water in the eastern United States and found that the∑PFASs median ranged from 2.2 to 40.0 ng/L, with the highest ∑PFAS value of 1645 ng/L. Regarding the contamination status of groundwater near a fluorochemical production plant, Petre et al. (2021) analyzed up to 29 PFASs in groundwater near a PFASs manufacturing facility and found that ∑PFAS in groundwater ranged from 20 − 4773 ng/L (mean = 1863 ng/L). Bräunig et al.^29^ investigated the presence of 10 PFASs near a fire-fighting training area, and the results showed that the compounds with a DF exceeding 50% included PFBA, PFPeA, PFHxA, PFOA and PFBS. The highest PFOS concentration measured in groundwater reached 13,000 ng/L, with an average of 4300 ng/L. In this study, the results obtained for the public supply wells were comparable to those obtained for areas without point sources. While, PFASs concentration measured in the domestic wells in this study was far higher than that reported in other regions except fire-fighting training areas in Australia.

Similar to the river water, groundwater samples were separated into two groups: group 1 (< 2 km) and group 2 (≥ 2 km). Geographical differences between two groups were evaluated (Fig. 2), and the findings indicated that samples collected in group 1 showed significantly high concentration of ∑PFASs (12073.1 ± 19017.9 ng/L, mean ± SD, *p* < 0.001), PFOS (4064.0 ± 11688.6 ng/L, *p* < 0.001), PFBA (3155.5 ± 2882.8 ng/L, *p* < 0.001), PFBS (1860.0 ± 2793.6 ng/L, *p* < 0.001) than those sampled in group 2 (∑PFASs: 294.4 ± 790.7 ng/L, PFOS: 192.0 ± 707.3 ng/L, PFBA: 36.5 ± 64.3 ng/L, PFBS: 19.8 ± 28.3 ng/L). Identifying the closed fluorochemical production plant as potentially important pollution sources of the nearby groundwater. These findings were in accordance with previous study conducted in France^30^, Spain^22^ and Australia^29^. Seasonal variations of PFASs concentrations in groundwater were also evaluated. The findings indicate that the levels of ∑PFASs (8364.9 ± 18435.8 ng/L, *p* < 0.01), PFOS (3644.8 ± 10822.8 ng/L, *p* < 0.01), PFBS (1164.2 ± 2593.7 ng/L, *p* < 0.01) and PFBA (1455.0 ± 2676.6 ng/L, *p* < 0.05) observed during dry periods were significantly higher compared to the wet season (∑PFASs: 2320.0 ± 4514.9 ng/L, PFOS: 58.1 ± 171.6 ng/L, PFBS: 452.6 ± 1179.4 ng/L, and PFBA: 1291.4 ± 2233.7 ng/L). Which is opposite to the seasonal trend of PFBA in surface water. McMahon et al.^28^ noted that there are many factors that may affect the occurrence of PFASs in groundwater, including land use, potential PFASs sources near the sampled wells, and hydrologic characteristics of groundwater systems. Compared to the river, groundwater systems are relatively confined and have slower flow velocities, thereby PFAS concentrations near point sources were found to be higher than those remote locations. And during wet seasons, surface water recharge mechanisms lead to observable decreases in PFASs levels. Nevertheless, as a result of the distinctive positioning of certain sample points, the seasonal trend at those sites varied from mentioned above. For example, ΣPFASs in W5 and W6 detected in wet season is higher than during the dry season. According to our survey, groundwater flows from southeast to northwest in the sampling area, and W5 is close to the PFASs source and located downstream, thus exhibiting a higher ΣPFASs value during the dry season.

The percentage of each substance at each groundwater sample point during the dry and wet seasons is shown in Fig. 3. Unlike the composition of species found in river water samples, the predominant PFASs species present in the groundwater are greatly influenced by both the geographical location and the season during which the samples were collected. In the four domestic wells (D2, D4, D1, and D3) in dry season, PFBS remains to be the predominant species of PFASs in D2 and D4, while PFHxS and PFOS are emerged as the dominant species in D1 and D3. These two PFASs species accounted for 55.4%, 51.1% and 26.0%, 32.0% of the total PFASs, indicating the potential presence of other PFASs sources in the surrounding area. In household wells, PFBA were the most prevalent (the proportion ranged from 44.5 to 70.2%) PFASs species during the wet season, while during dry season, the predominant PFASs species were PFOS and PFBA. As discussed above, when the erosive effects of rainwater are diminished in dry season, long-chain PFASs with high level of persistence^25,26^ tends to exhibited higher concentration in water, which is consistent with the results observed in river water. Nevertheless, the dominant PFASs congeners detected in groundwater, including PFBS, PFBA, PFHxS and PFOS, exhibited consistency with surface water, which are identified as hydrolysis products of the product of the fluorochemical manufacturing facility.

### Estimation of human exposure to PFASs

Multiple studies have demonstrated that general population can be exposed to PFASs through various pathway, such as the consumption of food^31^, drinking water^32^, and inhalation of air and dust^33^. While the dominant pathway responsible for human exposure to PFASs remains incompletely understood, drinking water is commonly recognized as an important contributor in overall intake.

**Fig. 4:**
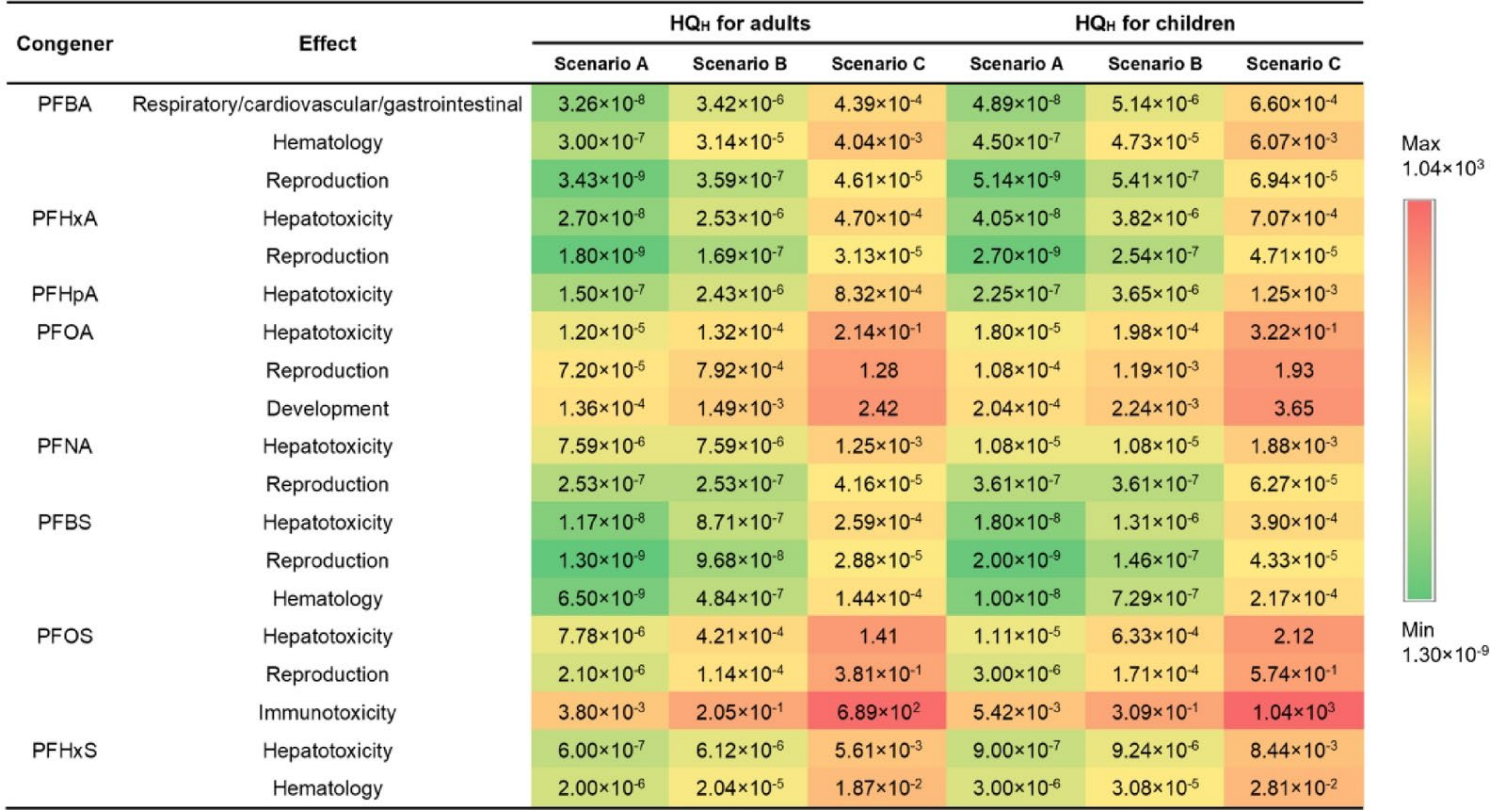
Estimated human health risk of individual PFASs in three exposure scenarios (**A**: low exposure, calculated with the minimum concentration detected; **B**: median exposure, calculated with the median concentration detected; **C**: high exposure, calculated with the maximum concentration detected for adults and children). Health risks from low to high were presented in color from green (min value) to yellow (median value) and to red (max value) according to their log-normalized value.

The exposure to the 8 most frequently detected PFASs (DF > 50% in both the public supply and domestic wells) via the consumption of drinking water was evaluated under scenarios A (low exposure, calculated with the minimum concentration detected), B (median exposure, calculated with the median concentration detected) and C (high exposure, calculated with the maximum concentration detected). The exposure doses of these 8 PFASs for adults were 0.1, 4.6 and 2318 ng/kg body weight (bw)/day under the three scenarios (Table S4). Toddlers are exposed approximately 50% greater than that experienced by adults, and their exposure doses were 0.1, 6.7, and 3488 ng/kg bw/day, respectively. The *TDI* value derived for the 8 PFASs ranged from 1.8 to 1.0 × 10^7^ ng/kg bw/day (Table S5). The potential health risks for adults and toddlers from exposure to individual PFASs under the low-exposure situation ranged from 1.3 × 10^−9^ to 3.8 × 10^−3^ and 2.0 × 10^−9^ to 5.4 × 10^−3^, respectively (Fig. 4, Table S6). The top health risks in both adults and children originated from PFOS due to its immunotoxicity characteristics, followed by PFOA due to its developmental, reproductive and liver toxicity characteristics. Under scenario B, the health risks of the 8 PFASs were one or two orders of magnitude greater compared to those under scenario A. Regarding the highest-risk congeners, the health risks of PFOS were 0.21 and 0.31 in adults and children, respectively. Under scenario C, the health risk further increased, with the health risks among adults and children ranging from 2.9 × 10^−5^ to 2.4 and 4.3 × 10^−5^ to 3.6, respectively. The top health risks for both adults and children still originated from PFOS due to its immunotoxicity, with health risk values as high as 6.9 × 10^2^ and 1.0 × 10^3^, respectively. Moreover, the adverse effects included the developmental toxicity of PFOA, with health risks for adults and children of 2.4 and 3.6, respectively, followed by the liver toxicity of PFOS and the reproductive toxicity of PFOA, with health risks for adults and children of 1.4 and 2.1 and 1.3 and 1.9, respectively. Overall, the health risks of PFOS and PFOA under scenario C far exceeded 1, suggesting that the health risks resulting from the consumption of drinking water were unacceptable and should not be ignored.

Qi et al.^34^ and Wei et al.^35^ reported that the health risks of PFOA and PFOS are far lower than 1 for all age groups resulting from the consumption of groundwater in nonindustrial areas in China. Zhang et al.^17^ estimated the human health risks of PFOS and PFOA in drinking water sources along the Yangtze River, China, and reported that the maximum hazard quotients were 0.029 and 0.043 for adults and children, respectively, under the worst-case scenario. In other countries and regions, such as the Ganges River basin in India^27^, Ronneby in Sweden^36^, and South Florida in the U.S^37^, the health risks of PFASs in drinking water were all below 1. By comparison, the health risk values in this study were significantly higher than those reported in the above studies, which may be related to the ground/drinking water sample locations in this study near the fluorochemical production plant. However, the health risks associated with PFOS and PFOA were much higher compared to the other congeners (the health risks of the other 6 frequently detected PFASs were all below 1), which is consistent with previous studies. Different countries and regions have proposed various guidelines based on human health to safeguard drinking water from PFASs contamination. For example, the Australian Government Department of Health has established a national standard for the sum levels of PFOS and PFHxS in drinking water, which should not exceed 70 ng/L^38^, and the U.S. Environmental Protection Agency has suggested a maximum contaminant level (MCL) of 4 ng/L for either PFOS or PFOA in drinking water^39^. In the drinking water quality standards for China, the limit values for PFOA and PFOS are set to 80 and 40 ng/L^40^, respectively. The maximum concentrations of PFOS and PFOA in domestic wells in this study far exceeded these guidelines, which also indicates that the PFOA and PFOS concentrations in groundwater near the fluorochemical production area pose a notable threat to the health of residents via the drinking water exposure pathway alone. In summary, it was essential to strengthen the management of PFAS emissions from farms, factories, and domestic sewage in the downstream to reduce the potential human health risks from groundwater PFAS. Besides, we also recommend enhanced monitoring of surface water and groundwater around fluorochemical production area to ensure drinking water safety. For contaminated drinking water, targeted removal measures, such as adsorption and filtration should be implemented to mitigate human exposure.

While this study only discussed HQ and did not calculate the Hazard Index (HI). And further research is required to more accurately assess the cumulative exposure risk associated with the combined toxicity of PFASs mixtures. Nevertheless, our findings highlight the critical need to prioritize PFASs monitoring in drinking water sources adjacent to fluorochemical manufacturing facilities, including those that have been decommissioned.

## Conclusion

This study focused on a fluorochemical production plant that used to be the largest POSF production facility in the world but stopped production in early 2021. The presence and human exposure to 17 PFASs in the surrounding ambient river and ground/drinking water within a 13 km around the facility were assessed. Out of the 17 analyzed PFASs, 11 were detected in river and ground/drinking water, with a total concentration ranging from 32.8 to 23,255 ng/L and RLs to 67937.9 ng/L, respectively. The dominant PFASs were those with shorter carbon chains (≤ 6 atoms), but the legacy compounds PFOA and PFOS also exhibited high concentrations. The spatial distribution indicated that the fluorochemical production plant resulted in high PFASs concentrations in the nearest river and ground water. Significant seasonal distributions differences in PFASs concentrations in the river and ground water were also observed. The main health risk for local residents (for the PFASs measured) stemmed from PFOS and PFOA. Under the high-exposure scenario, the hepatotoxicity, immunotoxicity and development toxicity risks of PFOS and PFOA all exceeded the safety limit. To control the risk of PFASs in drinking water, we strongly recommend that residents within 5 km of the facility should not utilize domestic well water as drinking water.

## Electronic supplementary material

Below is the link to the electronic supplementary material.

Supplementary Material 1

## Data Availability

The data that support the findings of this study are available from the corresponding authors upon reasonable request.
