# Supplementary material for: Occurrence and human exposure assessment of PFAS in river and groundwater around a closed fluorochemical plant in China
Source: Sci Rep. 2025 May 9;15:16241. doi: 10.1038/s41598-025-01128-6 (PMC12064658; doi:10.1038/s41598-025-01128-6)
Supplement: Supplementary file 1 — Supplementary Material 1 [file 41598_2025_1128_MOESM1_ESM.docx]

**Supplementary information**

**Occurrence and human exposure assessment of PFAS in river and groundwater around a closed fluorochemical plant in China**

Chunyan Xu^a,b^, Yijing Yang^a,b^, Haibo Ling^a,b^, Chuan Yi^a,b,^*, Xiangpu Zhang^a,b^, Ruowen Zhang^a,b^

^a^ Hubei Academy of Environmental Sciences, Wuhan, Hubei, People’s Republic of China,

^b^ Hubei key Laboratory of Pollution Damage Assessment and Environmental Health Risk Prevention and Control, Wuhan, Hubei, People’s Republic of China.

* Corresponding authors at: Hubei Academy of Environmental Sciences, Wuhan, Hubei, P.R. China.

*E-mail addresses*: yichuan0517@163.com.

**Table of contents**

| Table S1 | Characteristics of sampling sties | Page 3 |
| --- | --- | --- |
| Table S2 | Target analytes and optimized MS/MS parameters used for identifying and quantifying individual PFASs | Page 4 |
| Table S3 | Detection frequency (DF) and concentration of 17 PFASs | Page 5 |
| Table S4 | Human exposure through drinking to selected 8 PFASs congeners for adults and toddlers in three scenarios. | Page 5 |
| Table S5 | Calculated tolerable daily intake (TDI) of PFASs congeners. | Page 6 |
| Table S6 | Human health risk from PFASs on adults and children. | Page 7 |
| Reference |  | Page 8 |

**Table S1** Characteristics of sampling sties

| No. | Type | Latitude (N) | Longitude (E) | Straight distance to the point source (m) | Relative geographic location to the point source |
| --- | --- | --- | --- | --- | --- |
|  |  |  |  |  |  |
| UN1 | River water | 30.92137° | 113.57909° | 346.5 | Downstream |
| UN2 | River water | 30.93077° | 113.58201° | 721.6 | Upstream |
| UN3 | River water | 30.94592° | 113.58486° | 2430.8 | Upstream |
| FS1 | River water | 30.89997° | 113.57267° | 2823.9 | Downstream |
| FS2 | River water | 30.91628° | 113.56798° | 1452.8 | Downstream |
| FS3 | River water | 30.91979° | 113.55751° | 2191.1 | Downstream |
| FS4 | River water | 30.92677° | 113.55793° | 2110.2 | Upstream |
| W1 | Domestic well | 30.95785° | 113.54645° | 4893.4 | Downstream |
| W2 | Domestic well | 30.97369° | 113.57748° | 5495.8 | Downstream |
| W3 | Domestic well | 30.92597° | 113.54864° | 2989.5 | Downstream |
| W4 | Domestic well | 30.91886° | 113.56902° | 1609.0 | Downstream |
| W5 | Domestic well | 30.92106° | 113.57703° | 816.6 | Downstream |
| W6 | Domestic well | 30.92214° | 113.58352° | 184.8 | Downstream |
| W7 | Domestic well | 30.91490° | 113.58168° | 908.4 | Upstream |
| W8 | Domestic well | 30.90563° | 113.57541° | 2085.0 | Upstream |
| W9 | Domestic well | 30.92583° | 113.59447° | 951.0 | Upstream |
| W10 | Domestic well | 30.93413° | 113.59534° | 1603.0 | Downstream |
| D1 | Public supply well | 30.90421° | 113.66391° | 7771.0 | Upstream |
| D2 | Public supply well | 30.90931° | 113.71927° | 12892.0 | Upstream |
| D3 | Public supply well | 30.85631° | 113.65919° | 10102.0 | Upstream |
| D4 | Public supply well | 30.96694° | 113.65068° | 8003.0 | Upstream |

**Table S2** Target analytes and optimized MS/MS parameters used for identifying and quantifying individual PFASs

| Analyte | RT^a^ (min) | Precursor ion (m/z) | Product ion (m/z) | DP^b^ (V) | CE^c^ (V) |
| --- | --- | --- | --- | --- | --- |
| PFBA | 3.2 | 213 | 169 | -32 | -15 |
| PFPeA | 3.6 | 263 | 218.9 | -42 | -12 |
| PFHxA | 3.8 | 313 | 269 | -56 | -14 |
| PFHpA | 4 | 363 | 319/169 | -33 | -17/-25 |
| PFOA | 4.2 | 413 | 369.1/218.9 | -59 | -15/-23 |
| PFNA | 4.4 | 463 | 419.1/219 | -25 | -14/-25 |
| PFDA | 4.5 | 513 | 469/269 | -70 | -18/-22 |
| PFUnDA | 4.6 | 563 | 319/519 | -80 | -24/-17 |
| PFDoDA | 4.7 | 613.1 | 319/569 | -90 | -27/-19 |
| PFTrDA | 4.8 | 663.1 | 369/619 | -40 | -31.7/-19.6 |
| PFTeDA | 4.9 | 713.1 | 369/669 | -87 | -31/-19 |
| PFHxDA | 5.1 | 813.1 | 319/769 | -100 | -34/-21 |
| PFODA | 5.4 | 913.1 | 319/869 | -40 | -34/-27 |
| PFBS | 3.7 | 299 | 80/99 | -97 | -59/-37 |
| PFHxS | 4.1 | 399 | 80/99 | -133 | -74/-74 |
| PFOS | 4.4 | 498.9 | 80.1/99 | -120 | -88/-90 |
| PFDS | 4.6 | 599 | 80/99 | -180 | -117/-89 |

^a^RT, retention time; ^b^DP, declustering potential; ^c^CE, collision energy.

The first ion pair is used for quantification.

**Table S3** Detection frequency (DF^a^, %) and concentration (ng/L) of 17 PFASs (n=42)

| Analyte | River water (n = 14) | | | | | | Ground/drinking water (n = 28) | | | | |
| --- | --- | --- | --- | --- | --- | --- | --- | --- | --- | --- | --- |
|  | Min | max | mean | median | DF | Min | | max | mean | median | DF |
| PFBS | 10.3 | 11462.9 | 3436.9 | 206.5 | 100.0 | ND^b^ | | 9238.5 | 808.4 | 31.1 | 89.3 |
| PFHxS | 3.3 | 3549.0 | 975.4 | 29.5 | 100.0 | ND | | 5999.2 | 364.1 | 6.6 | 82.1 |
| PFPeA | 0.8 | 372.0 | 107.6 | 25.7 | 100.0 | ND | | 1259.8 | 135.7 | 4.8 | 78.6 |
| PFOS | 2.8 | 1392.8 | 543.7 | 362.2 | 100.0 | ND | | 40795.8 | 1851.4 | 12.2 | 75.0 |
| PFDS | ND | 8.3 | 1.3 | ND | 35.7 | ND | | 0.9 | ND | ND | 7.1 |
| PFBA | ND | 4789.8 | 1087.1 | 322.5 | 92.9 | ND | | 8639.5 | 1373.2 | 67.3 | 92.9 |
| PFHxA | 2.9 | 1462.7 | 419.5 | 109.7 | 100.0 | ND | | 3351.4 | 474.9 | 18.1 | 89.3 |
| PFHpA | 1.3 | 836.8 | 240.8 | 17.0 | 100.0 | ND | | 1779.8 | 126.9 | 3.2 | 82.1 |
| PFOA | 5.2 | 2333.9 | 585.3 | 27.1 | 100.0 | ND | | 4579.6 | 203.3 | 2.8 | 82.1 |
| PFNA | 0.3 | 10.6 | 3.9 | 2.0 | 100.0 | ND | | 37.0 | 2.0 | 0.2 | 64.3 |
| PFDA | ND | 2.5 | 0.5 | 0.2 | 50.0 | ND | | 52.9 | 2.5 | ND | 28.6 |
| PFUnDA | ND | ND | ND | ND | 0.0 | ND | | ND | ND | ND | 0.0 |
| PFDoDA | ND | ND | ND | ND | 0.0 | ND | | ND | ND | ND | 0.0 |
| PFTrDA | ND | 0.6 | ND | ND | 7.1 | ND | | 0.5 | ND | ND | 7.1 |
| PFTeDA | ND | ND | ND | ND | 0.0 | ND | | ND | ND | ND | 0.0 |
| PFHxDA | ND | ND | ND | ND | 0.0 | ND | | ND | ND | ND | 0.0 |
| PFODA | ND | ND | ND | ND | 0.0 | ND | | ND | ND | ND | 0.0 |

^a^DF: detection frequency. ^b^ND: defined as concentrations < RLs (0.1 ng/L).

**Table S4** Human exposure through drinking to selected 8 PFASs congeners for adults and toddlers in three scenarios.

| Congener | Adults Exposure (ng/kg bw/day) | | | Children Exposure (ng/kg bw/day) | | |
| --- | --- | --- | --- | --- | --- | --- |
|  | Scenario A | Scenario B | Scenario C | Scenario A | Scenario B | Scenario C |
| PFBA | 0.020 | 2.095 | 269.099 | 0.030 | 3.153 | 404.977 |
| PFHxA | 0.006 | 0.563 | 104.388 | 0.009 | 0.848 | 157.097 |
| PFHpA | 0.010 | 0.162 | 55.436 | 0.015 | 0.243 | 83.428 |
| PFOA | 0.008 | 0.088 | 142.643 | 0.012 | 0.132 | 214.669 |
| PFNA | 0.007 | 0.007 | 1.152 | 0.010 | 0.010 | 1.734 |
| PFBS | 0.013 | 0.968 | 287.757 | 0.020 | 1.457 | 433.055 |
| PFOS | 0.007 | 0.379 | 1270.689 | 0.010 | 0.570 | 1912.303 |
| PFHxS | 0.020 | 0.204 | 186.860 | 0.030 | 0.308 | 281.213 |
| Sum | 0.091 | 4.466 | 2318.024 | 0.136 | 6.721 | 3488.476 |

**Table S5** Calculated tolerable daily intake (TDI) of PFASs congeners.

| Congener | Species | Effect | Duration | Reference dose  ( mg/kg bw/day) | | Reference dose source | UFS | UFL | UFH | UFA | Calculated TDI  (×10^6^ ng/kg bw/day) |
| --- | --- | --- | --- | --- | --- | --- | --- | --- | --- | --- | --- |
| PFBA | Rat | Respiratory/cardiovascular/  gastrointestinal | Acute | NOAEL | 184 | ATSDR, 2021 | 10 | 1 | 3 | 10 | 6.1E+05 |
|  | Rat | Hematology | Subchronic | NOAEL | 6 | Butenhoff et al., 2012a | 3 | 1 | 3 | 10 | 6.7E+04 |
|  | Mouse | Reproduction | One generation | NOAEL | 175 | Das et al., 2008 | 1 | 1 | 3 | 10 | 5.8E+06 |
| PFHxA | Rat | Hepatotoxicity | Subchronic | NOAEL | 20 | Loveless et al., 2009 | 3 | 1 | 3 | 10 | 2.2E+05 |
|  | Mouse | Reproduction | One generation | NOAEL | 100 | Iwai and Hoberman, 2014 | 1 | 1 | 3 | 10 | 3.3E+06 |
| PFHpA | Mouse | Hepatotoxicity | Acute | NOAEL | 20 | Kudo et al., 2006 | 10 | 1 | 3 | 10 | 6.7E+04 |
| PFOA | Rat | Hepatotoxicity | Subchronic | NOAEL | 0.06 | Perkins et al., 2004 | 3 | 1 | 3 | 10 | 6.7E+02 |
|  | Mouse | Reproduction | One generation | LOAEL | 0.01 | Macon et al., 2011 | 1 | 3 | 3 | 10 | 1.1E+02 |
|  | Mouse | Development | One generation | LOAEL | 0.0053 | Minnesota Department of Health, 2020 | 1 | 3 | 3 | 10 | 5.9E+01 |
| PFNA | Mouse | Hepatotoxicity | Subacute | LOAEL | 0.83 | Wolf et al., 2010 | 10 | 3 | 3 | 10 | 9.2E+02 |
|  | Mouse | Reproduction | One generation | NOAEL | 0.83 | Wolf et al., 2010 | 1 | 1 | 3 | 10 | 2.8E+04 |
| PFBS | Rat | Hepatotoxicity | Subchronic | NOAEL | 100 | Lieder et al., 2009 | 3 | 1 | 3 | 10 | 1.1E+06 |
|  | Rat | Reproduction | One generation | NOAEL | 300 | Lieder et al., 2009 | 1 | 1 | 3 | 10 | 1.0E+07 |
|  | Rat | Hematology | Subchronic | NOAEL | 60 | Minnesota Department of Health, 2011 | 1 | 1 | 3 | 10 | 2.0E+06 |
| PFOS | Rat | Hepatotoxicity | Chronic | NOAEL | 0.027 | Butenhoff et al., 2012b | 1 | 1 | 3 | 10 | 9.0E+02 |
|  | Rat | Reproduction | One generation | NOAEL | 0.1 | Organisation for Economic Co-operation and Development, 2002 | 1 | 1 | 3 | 10 | 3.3E+03 |
|  | Mouse | Immunotoxicity | subacute | LOAEL | 0.0017 | Peden-Adams et al., 2008 | 10 | 3 | 3 | 10 | 1.8E+00 |
| PFHxS | Mouse | Hepatotoxicity | Acute | NOAEL | 10 | Das et al., 2017 | 10 | 1 | 3 | 10 | 3.3E+04 |
|  | Rat | Hematology | Subchronic | NOAEL | 3 | Butenhoff et al., 2009 | 10 | 1 | 3 | 10 | 1.0E+04 |

**Table S6** Human health risk from PFASs on adults and children.

| Congener | Effect | HR_S_ for adults | | | HR_S_ for children | | |
| --- | --- | --- | --- | --- | --- | --- | --- |
|  |  | Scenario A | Scenario B | Scenario C | Scenario A | Scenario B | Scenario C |
| PFBA | Respiratory/cardiovascular/  gastrointestinal | 3.3E-09 | 3.4E-06 | 4.4E-04 | 4.9E-08 | 5.1E-06 | 6.6E-04 |
|  | Hematology | 3.0E-07 | 3.1E-05 | 4.0E-03 | 4.5E-07 | 4.7E-05 | 6.1E-03 |
|  | Reproduction | 3.4E-09 | 3.6E-07 | 4.6E-05 | 5.1E-09 | 5.4E-07 | 6.9E-05 |
| PFHxA | Hepatotoxicity | 2.7E-08 | 2.5E-06 | 4.7E-04 | 4.1E-08 | 3.8E-06 | 7.1E-04 |
|  | Reproduction | 1.8E-09 | 1.7E-07 | 3.1E-05 | 2.7E-09 | 2.5E-07 | 4.7E-05 |
| PFHpA | Hepatotoxicity | 1.5E-07 | 2.4E-06 | 8.3E-04 | 2.3E-07 | 3.6E-06 | 1.3E-03 |
| PFOA | Hepatotoxicity | 1.2E-05 | 1.3E-04 | 2.1E-01 | 1.8E-05 | 2.0E-04 | 3.2E-01 |
|  | Reproduction | 7.2E-05 | 7.9E-04 | 1.3E+00 | 1.1E-04 | 1.2E-03 | 1.9E+00 |
|  | Development | 1.4E-04 | 1.5E-03 | 2.4E+00 | 2.0E-04 | 2.2E-03 | 3.6E+00 |
| PFNA | Hepatotoxicity | 7.6E-06 | 7.6E-06 | 1.2E-03 | 1.1E-05 | 1.1E-05 | 1.9E-03 |
|  | Reproduction | 2.5E-07 | 2.5E-07 | 4.2E-05 | 3.6E-07 | 3.6E-07 | 6.3E-05 |
| PFBS | Hepatotoxicity | 1.2E-08 | 8.7E-07 | 2.6E-04 | 1.8E-08 | 1.3E-06 | 3.9E-04 |
|  | Reproduction | 1.3E-09 | 9.7E-08 | 2.9E-05 | 2.0E-09 | 1.5E-07 | 4.3E-05 |
|  | Hematology | 6.5E-09 | 4.8E-07 | 1.4E-04 | 1.0E-08 | 7.3E-07 | 2.2E-04 |
| PFOS | Hepatotoxicity | 7.8E-06 | 4.2E-04 | 1.4E+00 | 1.1E-05 | 6.3E-04 | 2.1E+00 |
|  | Reproduction | 2.1E-06 | 1.1E-04 | 3.8E-01 | 3.0E-06 | 1.7E-04 | 5.7E-01 |
|  | Immunotoxicity | 3.8E-03 | 2.1E-01 | 6.9E+02 | 5.4E-03 | 3.1E-01 | 1.0E+03 |
| PFHxS | Hepatotoxicity | 6.0E-07 | 6.1E-06 | 5.6E-03 | 9.0E-07 | 9.2E-06 | 8.4E-03 |
|  | Hematology | 2.0E-06 | 2.0E-05 | 1.9E-02 | 3.0E-06 | 3.1E-05 | 2.8E-02 |

**Reference**

Agency for Toxic Substances and Disease Registry (ATSDR), 2021. Toxicological profile for Perfluoroalkyls. Atlanta, GA: U.S. Department of Health and Human Services, Public Health Service. http://doi.10.15620/cdc:59198

Butenhoff J.L., Bjork J.A., Chang S.C., Ehresman D.J., Parker G.A., Das K., Lau C., Lieder P.H., van Otterdijk F.M., Wallace K.B., 2012a. Toxicological evaluation of ammonium perfluorobutyrate in rats: Twenty-eight-day and ninety-day oral gavage studies. Reprod. Toxicol. 33, 513-530. http://doi.10.1016/j.reprotox.2011.08.004.

Butenhoff J.L., Chang S.C., Ehresman D.J., York R.G., 2009. Evaluation of potential reproductive and developmental toxicity of potassium perfluorohexanesulfonate in Sprague Dawley rats. Reprod. Toxicol. 27, 331-341. http://doi.10.1016/j.reprotox.2009.01.004.

Butenhoff J.L., Chang S.C., Olsen G.W., Thomford P.J., 2012b. Chronic dietary toxicity and carcinogenicity study with potassium perfluorooctanesulfonate in Sprague Dawley rats. Toxicology 293, 1-15. <http://doi.10.1016/j.tox.2012.01.003>.

Das K.P., Grey B.E., Zehr R.D., Wood C.R., Butenhoff J.L., Chang S.C., Ehresman D.J., Tan Y.M., Lau C., 2008. Effects of perfluorobutyrate exposure during pregnancy in the mouse. Toxicol. Sci. 105, 173-181. http://doi.10.1093/toxsci/kfn099.

Das K.P., Wood C.R., Lin M.M.T., Starkov A.A., Lau C., Wallace K.B., Corton J.C., Abbott B.D., 2017. Perfluoroalkyl acids-induced liver steatosis: Effects on genes controlling lipid homeostasis. Toxicology 378, 37-52. http://doi.10.1016/j.tox.2016.12.007.

Iwai H., Hoberman A.M., 2014. Oral (Gavage) Combined Developmental and Perinatal/Postnatal Reproduction Toxicity Study of Ammonium Salt of Perfluorinated Hexanoic Acid in Mice. Int. J. Toxicol. 33, 219-237. http://doi.10.1177/1091581814529449.

Kudo N., Suzuki-Nakajima E., Mitsumoto A., Kawashima Y., 2006. Responses of the liver to perfluorinated fatty acids with different carbon chain length in male and female mice: In relation to induction of hepatomegaly, peroxisomal beta-oxidation and microsomal 1-acylglycerophosphocholine acyltransferase. Biol. Pharm. Bull. 29, 1952-1957. http://doi.10.1248/bpb.29.1952.

Lieder P.H., York R.G., Hakes D.C., Chang S.C., Butenhoff J.L., 2009. A two-generation oral gavage reproduction study with potassium perfluorobutanesulfonate (K+PFBS) in Sprague Dawley rats. Toxicology 259, 33-45. http://doi.10.1016/j.tox.2009.01.027.

Loveless S.E., Slezak B., Serex T., Lewis J., Mukerji P., O'Connor J.C., Donner E.M., Frame S.R., Korzeniowski S.H., Buck R.C., 2009. Toxicological evaluation of sodium perfluorohexanoate. Toxicology 264, 32-44. http://doi.10.1016/j.tox.2009.07.011.

Macon M.B., Villanueva L.R., Tatum-Gibbs K., Zehr R.D., Strynar M.J., Stanko J.P., White S.S., Helfant L., Fenton S.E., 2011. Prenatal Perfluorooctanoic Acid Exposure in CD-1 Mice: Low-Dose Developmental Effects and Internal Dosimetry. Toxicol. Sci. 122, 134-145. <http://doi.10.1093/toxsci/kfr076>.

Minnesota Department of Health, 2011. 2011 Health Risk Limits for Groundwater. https://www.health.state.mn.us/communities/environment/risk/docs/guidance/gw/pfbs.pdf

Minnesota Department of Health, 2020. Toxicological Summary for: Perfluorooctanoate. <https://www.health.state.mn.us/communities/environment/risk/docs/guidance/gw/pfoa.pdf>

Organisation for Economic Co-operation and Development, 2002. HAZARD ASSESSMENT OF PERFLUOROOCTANE SULFONATE (PFOS) AND ITS SALTS. https://www.oecd.org/chemicalsafety/risk-assessment/2382880.pdf

Peden-Adams M.M., Keller J.M., EuDaly J.G., Berger J., Gilkeson G.S., Keil D.E., 2008. Suppression of humoral immunity in mice following exposure to perfluorooctane sulfonate. Toxicol. Sci. 104, 144-154. http://doi.10.1093/toxsci/kfn059.

Perkins R.G., Butenhoff J.L., Kennedy G.L., Palazzolo M.J., 2004. 13-week dietary toxicity study of ammonium perfluorooctanoate (APFO) in male rats. Drug Chem. Toxicol. 27, 361-378. <http://doi.10.1081/dct-200039773>.

Wolf C.J., Zehr R.D., Schmid J.E., Lau C., Abbott B.D., 2010. Developmental Effects of Perfluorononanoic Acid in the Mouse Are Dependent on Peroxisome Proliferator-Activated Receptor-Alpha. PPAR Res. 2010, 11. http://doi.10.1155/2010/282896.
